# What outcomes do studies use to measure the impact of prognostication on people with advanced cancer? Findings from a systematic review of quantitative and qualitative studies

**DOI:** 10.1177/02692163231191148

**Published:** 2023-08-10

**Authors:** Caitlin Spooner, Bella Vivat, Nicola White, Andrea Bruun, Gudrun Rohde, Pei Xing Kwek, Patrick Stone

**Affiliations:** 1 Marie Curie Palliative Care Research Department, University College London, London, UK; 2 Faculty of Health and Sport Sciences, University of Agder, Kristiansand, Norway; 3 University College Dublin School of Medicine, University College Dublin, Dublin, Ireland

**Keywords:** Palliative care, prognosis, neoplasms, adult, caregivers, outcome assessment (health care), systematic review

## Abstract

**Background:**

Studies evaluating the impact of prognostication in advanced cancer patients vary in the outcomes they measure, and there is a lack of consensus about which outcomes are most important.

**Aim:**

To identify outcomes previously reported in prognostic research with people with advanced cancer, as a first step towards constructing a core outcome set for prognostic impact studies.

**Design:**

A systematic review was conducted and analysed in two subsets: one qualitative and one quantitative. (PROSPERO ID: CRD42022320117; 29/03/2022).

**Data sources:**

Six databases were searched from inception to September 2022. We extracted data describing (1) outcomes used to measure the impact of prognostication and (2) patients’ and informal caregivers’ experiences and perceptions of prognostication in advanced cancer. We classified findings using the Core Outcome Measures in Effectiveness Trials (COMET) initiative taxonomy, along with a narrative description. We appraised retrieved studies for quality, but quality was not a basis for exclusion.

**Results:**

We identified 42 eligible studies: 32 quantitative, 6 qualitative, 4 mixed methods. We extracted 70 outcomes of prognostication in advanced cancer and organised them into 12 domains: (1) survival; (2) psychiatric outcomes; (3) general outcomes; (4) spiritual/religious/existential functioning/wellbeing, (5) emotional functioning/wellbeing; (6) cognitive functioning; (7) social functioning; (8) global quality of life; (9) delivery of care; (10) perceived health status; (11) personal circumstances; and (12) hospital/hospice use.

**Conclusion:**

Outcome reporting and measurement varied markedly across the studies. A standardised approach to outcome reporting in studies of prognosis is necessary to enhance data synthesis, improve clinical practice and better align with stakeholders’ priorities.

**What is already known about the topic?**

- Currently, there is no gold standard for evaluating how different methods of prognosticating in advanced cancer impact on patient care.
- Prognostic models are principally evaluated by their statistical performance, determining their discrimination and calibration. However, before any prognostic model can be recommended for use in clinical practice, it is necessary to demonstrate whether or not it has a beneficial impact on patient care.
- There is a lack of consensus among stakeholders about how to assess the impact of prognostication in advanced cancer, with prognostic studies varying in the outcomes they select.

**What this paper adds?**

- We identified a wide variety of outcomes and measures used in published studies, which makes inter-study comparability problematic.
- Our findings highlight the widespread effect that prognostication in advanced cancer has on patients and informal caregivers.
- The lived experiences of patients and informal caregivers regarding prognostication in advanced cancer are not always represented in the outcomes quantitative prognostic studies measure.

**Implications for practice, theory or policy**

- Further research is needed to identify and prioritise outcomes to measure the impact of prognostication in advanced cancer.
- Patients’ and informal caregivers’ experiences and perspectives should always be incorporated when evaluating the impact of prognostication.
- Outcome selection in prognostication studies needs to be more consistent and standardised.

## Introduction

Prognostication of a person’s likely length of life is a vital component of palliative care, patient care and decision-making.^1,2^ Accurate prognoses aim to provide patients, their families and informal caregivers with sufficient time to prepare for the end-of-life such as making financial plans and identifying their preferences for place of death.^3^ Accurate prognoses also enable clinicians to identify appropriate treatment strategies based on individual patient's prognostic factors and symptoms.^2^ Conversely, inaccurate prognoses of end-of-life can damage patients' psychological wellbeing and sense of hope.^4–6^

No method of prognostication is completely accurate. In daily practice, prognostication about end-of-life is often a clinical estimate based on clinicians’ skills and experiences.^3^ However, clinicians' estimates are often inaccurate, inconsistent and over-optimistic.^7,8^ Various prognostic tools have been validated for use in people with advanced cancer, but none has yet demonstrated clearly superior discrimination, calibration or accuracy over clinicians’ predictions.^3^ It is possible that methods of prognostication may vary in other respects, such as ease of use or interpretation. However, the comparative impact of different prognostic methods has yet to be established, and variation in outcome reporting among studies makes it difficult to distinguish the impact of different methods of prognosticating with similar levels of accuracy.

Developing a standardised set of outcomes is integral to improving the consistency of reporting of outcomes, inter-trial comparisons and informing clinical decision-making in the context of prognostication in advanced cancer.^9^ Such guidance for outcome reporting in prognostic studies does not exist, and no systematic review has been conducted to explore the extent of variation in outcomes reported in prognostic studies. Our review aimed to identify studies reporting on all outcomes or experiences of prognostication in advanced cancer, as a first step in developing a core outcome set for use in future prognostic impact studies. We sought therefore to include the broadest possible range of studies, so as not to pre-emptively exclude any potentially relevant outcomes in advance of testing with stakeholders.

We analysed the results of this systematic review in two subsets, exploring: (1) quantitative studies of the impact of end-of-life prognostication in advanced cancer; (2) qualitative studies of patient and informal caregiver experiences and perspectives.

The main review question was:

- What outcomes for end-of-life prognostication in advanced cancer have been identified in research studies to date?

The subsidiary review questions were:

- What quantitative outcomes are measured in studies of advanced cancer where survival estimates are provided?
- What are patients and informal caregivers’ qualitative experiences and perceptions of prognostication in advanced cancer?

## Methods

This systematic review was conducted according to the Cochrane Handbook for Systematic Reviews of Interventions^10^ and reported as per the Preferred Reporting Items for Systematic Reviews and Meta-Analyses (PRISMA) guidelines.^11^ The protocol was registered prospectively on the International Prospective Register of Systematic Reviews (PROSPERO) (CRD42022320117, 29/03/2022, https://www.crd.york.ac.uk/prospero/display_record.php?ID=CRD42022320117).

We were aware that papers reporting on studies of populations relevant to our study aim might be heterogeneous in the populations they included. We therefore formulated a term and a working definition to encompass all relevant populations, so as to ensure that all papers with potentially relevant participants were included, and none excluded due to an over-narrow definition. Informed by previously published papers,^12–18^ we decided on the term ‘advanced cancer patients’, meaning individuals diagnosed with a cancer in the advanced stages (metastatic, locally advanced or recurrent). Studies including any patients who were receiving treatment or care without curative intent (i.e. with the objective being to improve symptoms and quality of life, and/or slow disease progression, but not to cure the cancer) were eligible for inclusion.

### Eligibility criteria

Eligibility criteria are shown in Table 1. Papers were screened for study type rather than using limits to restrict the results. Where only abstracts were found, we contacted authors for a published article.

**Table 1.** Eligibility criteria.

PICOS criteria	Inclusion criteria	Exclusion criteria
Population	• Aged ⩾18 years	• Aged <18 years
	• Advanced cancer patients	• Cancer survivors, healthy participants or the general population
	• Informal caregivers of advanced cancer patients	
Intervention	• Prognostication, defined as any process of estimating and communicating the length of survival of an individual’s disease	• Interventions similar to prognostication, such as advance care planning, early palliative care planning, goals of care or communication skills training
		• Prognosis was given in response to an intervention with curative intent (e.g. surgery, chemotherapy, radiotherapy or a biological therapy where the prognosis would be dependent on their response to that treatment (e.g. probability of surviving surgery or having a complete response to chemotherapy)
		• Studies that aimed to validate prognostic scores, factors (such as biomarkers) or values
Comparison	• Any comparator, including those who received no prognosis information or no comparator, with a single cohort of participants.	• Not applicable
Outcome	• Outcomes used to measure the impact of prognostication, including but not limited to clinical, symptomatic and psychological outcomes, as well as service-level outcomes such as waiting lists and bed occupancy	• Traditional measures of prognostic accuracy, which is statistical tests to evaluate prognostic accuracy (*C*-statistics; ROC curves).
	• Patient and informal caregivers’ experiences and perceptions of prognostication.	
Study	• English language	• Studies published in languages other than English
	• Original quantitative studies^a^: randomised/quasi-randomised controlled trials, interventional studies and observational studies (cohort, cross-sectional and case-control studies)	• Systematic reviews, diagnostic studies, editorials, commentaries, review articles, case reports and letters without original data
	• Qualitative studies (including interviews, focus groups and qualitative observation)	• Conference abstracts for which authors were unable to provide full data

a Mixed method studies were only incorporated if the qualitative and quantitative components were reported independently and where the relevant data could be extracted.

We piloted the screening process prior to full data extraction. The piloting confirmed that studies’ descriptions of their samples varied considerably. We therefore decided that, for studies to be eligible for inclusion, if papers did not specify that the population was in the advanced stages of disease, the sample description should include one or more of the following:

- Life-limiting
- Terminal
- Palliative
- End-of-life
- Deceased, where cancer was the primary cause of death
- Connected to a palliative care team, service, unit or hospice
- Predicted prognosis of ⩽12 months or a ‘poor’ prognosis
- Survival time at most 9 months

### Information sources

MEDLINE, EMBASE, PsycINFO, CINAHL, the Cochrane Controlled Register of Trials and the Cochrane Central Register were searched from inception to September 2022. Search limits were applied to restrict results to a human, adult population and studies published in English language only due to limitations in resources.

The search included trials registered at ClinicalTrials.gov and The International Clinical Trials Registry Platform Search Portal. Grey literature searches were conducted using OpenGrey and ProQuest-Digital Dissertations and Theses. Finally, study references of included studies were searched forward and backward.

### Search strategy

Keywords related to prognostication, palliative care, advanced cancer and outcomes and cognate terms were included in the search strategy (Supplemental Appendix 1).

### Selection process

Search results were imported into the software *Rayyan*^19^ and de-duplicated. Four authors (CS, AB, GR and PXK) independently screened titles and abstracts against eligibility criteria. Those that met the inclusion criteria for either review question were read in full by at least two authors. Discrepancies were resolved through discussion between authors. If no consensus could be reached, a third author was consulted.

### Data collection process and data items

Four authors (CS, AB, GR and PXK) extracted key information (author/s, year of publication, geographical setting, methodology, sample size, population, study setting, data collection methods, data describing outcomes of prognostication) from eligible studies using a data extraction form (Supplemental Appendix 2) designed by CS and piloted by four authors (CS, AB, GR and PXK) on a random sample of five studies before being fully implemented.

### Study quality assessment

The Mixed Methods Appraisal Tool (MMAT) was used to assess the quality of included studies.^20^ This tool is useful for systematic reviews of studies of mixed methodology because it enables critical appraisal of all study methodologies, including randomised and nonrandomised.^20^ It also includes criteria for critically appraising mixed methods studies, which are lacking in other tools. For each included study, the authors chose the appropriate category of studies (qualitative, quantitative or mixed methods) and rated the criteria of the chosen category using the following responses: ‘yes’, ‘no’ or ‘can’t tell’. The number of ‘yes’ responses for each study were aggregated, to provide an overview of the scores for methodological quality of the current literature. The aim of the review was to comprehensively identify and synthesise outcomes reported in extant studies; therefore, we did not exclude studies on the basis of quality.

### Synthesis methods

We anticipated heterogeneity of included studies and therefore decided that a narrative synthesis was the most appropriate method for synthesising findings. We summarised study characteristics using descriptive statistics.

A table of quantitative outcomes was generated, including descriptions of outcomes, measures and frequency of reporting. Outcomes were categorised using the taxonomy for core outcome sets recommended by COMET.^21^ This taxonomy consists of five core areas: (1) death; (2) physiological/clinical; (3) life impact; (4) resource use and (5) adverse events. A narrative description was provided for each outcome.

Relevant findings from qualitative studies are not necessarily presented in ways which translate directly to the COMET taxonomy. We therefore identified themes from these qualitative study findings, which we then categorised by COMET areas and domains. CS performed a thematic synthesis using NVivo version 11,^22^ extracting data from verbatim quotes provided in the included studies and supporting author interpretations. The synthesis had three stages:^23^ (1) coding data pertaining to patient and informal caregiver experiences and perceptions of prognostication; (2) grouping codes into descriptive themes and (3) developing analytical themes using the COMET taxonomy^21^ as a framework, to enable later combination of results of both sets of studies. We produced a narrative description of each theme in relation to the review question, to support our thematic analysis and allocation of themes to COMET taxonomy areas and domains. Any disparities or discrepancies that arose at any stages of thematic synthesis were resolved through discussion and consensus with other authors.

## Results

### Study selection

**Figure 1.**
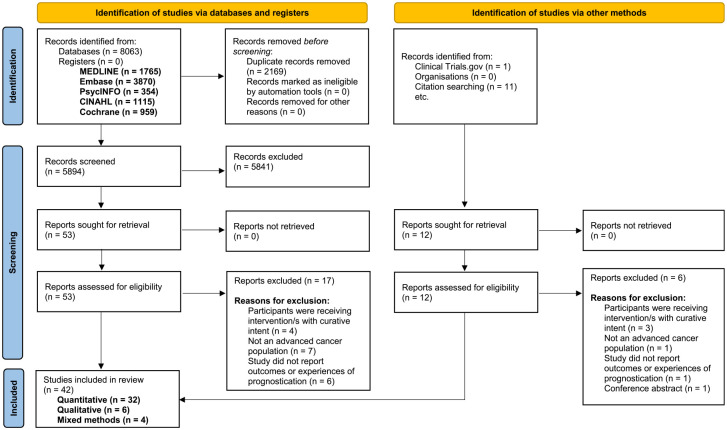
PRISMA flow diagram.

The search returned 8075 results. Following de-duplication, we screened 5906 records by title and abstract against the eligibility criteria, and 65 of these were potentially eligible. After reading these articles in full-text, 42 fulfilled the inclusion criteria: 32 quantitative, 6 qualitative and 4 mixed-methods. Figure 1 summarises the study selection process.^11^

### Study characteristics

Table 2 summarises the characteristics of included studies. Studies most frequently came from the United States of America (USA; *n* = 13) and Taiwan (*n* = 9). Sample sizes ranged from 14 to 3445. Many studies (*n* = 27) included patients only, 8 included patients and informal caregivers and 7 included informal caregivers only.

**Table 2.** Characteristics of included studies.

Authors (year)	Geographical setting	Study design (methodology)	Sample size	Sample description	Study setting	Data collection method	Data for analysis	
							Quantitative	Qualitative
Aabom et al.^24^	Denmark	Cohort study (retrospective)	3445	Deceased patients where cancer (any) was the primary cause of death	Danish Cancer Register	Registry data	X	
Ahn et al.^27^	South Korea	Cross-sectional study	345	Advanced cancer patients (any)	34 palliative care services	Surveys	X	
An et al.^36^	South Korea	Cohort study (prospective)	718 (359 dyads)	Terminal cancer patients (any) and caregivers	12 hospitals	Interviews, medical records and questionnaires	X	
Applebaum et al^62^	USA	Mixed methods	64 (32 dyads)	Cancer patients (MG) defined as being incurable and caregivers	1 cancer centre	Questionnaires and semi-structured interviews		X
Baek et al.^37^	South Korea	Cohort study (prospective)	98	Advanced cancer patients (any)	2 hospitals; 1 cancer centre	Medical records and questionnaires	X	
Barnett^61^	UK	Mixed methods	106	Advanced cancer patients (any)	Unknown number of oncology centres, hospitals and general practices	Questionnaires, semi-structured and open-ended interviews	X	X
Bradley et al^65^	USA	Cross-sectional study	232	Terminal cancer patients (brain, pancreas, liver, gall bladder or inoperable lung)	6 hospitals	Medical records	X	
Chan^28^	Hong Kong	Cross-sectional study	935	Cancer patients (any) in a palliative care unit	1 hospital	Medical records	X	
Chen et al^29^	Taiwan	RCT	920 (460 dyads)	Terminal cancer patients (any) and caregivers	1 medical centre	Medical records	X	
Cherlin et al^63^	USA	Mixed methods	218	Caregivers of cancer patients (any) enrolled in a hospice	1 hospice	Questionnaires, open-ended and in-depth interviews		X
Chochinov et al^44^	Canada	Cross-sectional study	200	Terminal cancer patients (any)	2 hospitals	Interviews and survey	X	
Clayton et al.^59^	Australia	Not stated	43	Terminal cancer patients (any) and caregivers	3 palliative care services	Focus groups and semi-structured interviews		X
Fenton et al.^30^	USA	Cohort study (longitudinal)	265	Advanced cancer patients (stage IV non-haematological or stage III any)	4 cancer clinics; 3 medical centres; 3 hospitals	Surveys	X	
Friedrichsen et al.^55^	Sweden	Hermeneutic approach	45	Terminal cancer patients (any)	2 hospital-based home care units	Semi-structured interviews		X
Gramling et al.^31^	USA	Cohort study (prospective)	230	Advanced cancer patients (any)	2 medical centres	Questionnaires	X	
Gramling et al.^38^	USA	Cohort study (prospective)	231	Advanced cancer patients (any)	2 medical centres	Questionnaires	X	
Helft et al^45^	USA	Mixed methods	179	Advanced cancer patients (any)	1 university	Interviews (using both quantitative and qualitative methods)	X	
Janssens et al.^39^	Belgium	Cross-sectional study	125	Advanced cancer patients (lung)	10 hospitals	Questionnaires	X	
Kao et al.^46^	Taiwan	Cohort study (retrospective)	2995	Terminal cancer patients (any)	1 medical centre	Interviews and medical records	X	
Kim et al.^32^	South Korea	Cohort study (prospective)	262	Terminal cancer patients (any)	1 palliative care unit	Interviews and questionnaires	X	
Kirk et al.^56^	Australia and Canada	Not stated	72	Cancer patients (any) registered with a palliative care programme and caregivers	2 hospices	Semi-structured interviews		X
Lambden et al.^47^	USA	Longitudinal study (prospective)	85	Advanced cancer patients (any)	7 outpatient cancer clinics	Interviews and medical records	X	
Lee et al.^40^	South Korea	Cohort study (prospective)	98	Terminal cancer patients (any)	2 hospitals	Interviews; medical records and questionnaires	X	
Lundquist et al.^48^	Sweden	Cohort study (retrospective)	2382	Deceased patients where cancer (any) was the primary cause of death	Swedish Register for Palliative Care	Registry data	X	
Nielsen et al.^41^	Denmark	Cohort study (prospective)	2865	Caregivers of terminal cancer patients	Danish Medicines Agency	Questionnaires	X	
Nipp et al.^33^	USA	Cross-sectional study	350	Cancer patients (lung or GI) defined as being incurable	1 hospital	Questionnaires	X	
Park et al.^60^	USA	Exploratory	244	Caregivers of deceased patients where cancer (any) was the primary cause of death	Open-access, educational website	Open-ended survey questions		X
Park et al.^42^	USA	Cross-sectional study	344	Caregivers of deceased patients where cancer (any) was the primary cause of death	Open-access, educational website	Survey	X	
Ray et al.^25^	USA	Longitudinal (prospective)	560 (280 dyads)	Terminal cancer patients (any) and caregivers	4 cancer centres	Interviews	X	
Røen et al.^57^	Norway	Exploratory	14	Caregivers of advanced cancer patients (any)	2 outpatient palliative care/cancer clinics	Semi-structured interviews		X
Shen et al.^49^	USA	Longitudinal (prospective)	558 (279 dyads)	Advanced cancer patients (any) and caregivers	11 oncology outpatient sites	Interviews	X	
Sudhakar et al.^58^	India	Phenomenological approach	208	Caregivers of deceased patients where cancer (any) was the primary cause of death	1 cancer centre	Open-ended interviews		X
Tang et al.^43^	Taiwan	Cross-sectional study	2216 (1108 dyads)	Terminal cancer patients (any) and caregivers	24 hospitals	Interviews and questionnaires	X	
Tang et al.^34^	Taiwan	Cross-sectional study	2452	Terminal cancer patients (any)	23 hospitals	Interviews and medical records	X	
Tang et al.^50^	Taiwan	Longitudinal (prospective)	249	Terminal cancer patients (any)	1 medical centre	Interviews	X	
Tang et al.^51^	Taiwan	Longitudinal (prospective)	325	Terminal cancer patients (any)	1 medical centre	Interviews and questionnaires	X	
Tang et al.^52^	Taiwan	Longitudinal (prospective)	256	Terminal cancer patients (any)	1 medical centre	Interviews	X	
Tang et al.^53^	Taiwan	Longitudinal (prospective)	277	Terminal cancer patients (any)	1 medical centre	Interviews	X	
Thompson et al.^64^	Canada	Cross-sectional study	381	Advanced cancer patients (any)	Inpatient palliative care units or hospices, hospitals or home care across 8 communities in Canada	Interviews	X	
Vlckova et al.^26^	Czech Republic	Cross-sectional study	129	Advanced cancer patients (any)	3 hospitals	Structured patient interviews	X	
Wen et al.^54^	Taiwan	Longitudinal (prospective)	218	Terminal cancer patients (any)	1 medical centre	Patient interviews	X	
Yoshida et al.^35^	Japan	Cross-sectional study	666	Caregivers of cancer patients (any) admitted to palliative care units	100 palliative care units	Questionnaire	X	

GI: Gastrointestinal; MG: malignant glioma; RCT: randomised controlled trial; UK: United Kingdom; USA: United States of America.

### Methodological quality

We assessed the quality of the 32 quantitative studies as high in 12 studies,^24–35^ moderate in 9,^36–43^ and low in 11.^44–54^ We assessed the quality of the six qualitative studies as high in four,^55–58^ and moderate in two.^59, 60^ We assessed the quality of the four mixed methods studies as moderate in one,^61^ and low in the remaining three.^62–64^ The quality assessments are summarised in Supplemental Appendix 3.

### Results of quantitative synthesis

Of 42 studies identified, 34 reported on outcomes used to measure impact of prognostication in advanced cancer.^24–40,42–54,61,64–66^ From quantitative data, 58 outcomes of prognostication were identified. Table 3 lists identified outcomes.

**Table 3.** Outcomes and measures of prognostication in advanced cancer identified in the quantitative data, according to the core areas and domains within the COMET taxonomy.

Core area	Domain	Outcome	Measure	No. of studies reporting data on this outcome (*n* = 34)
Death	Survival	Length of survival (days)	Medical records and death certificates	4
Physiological/clinical outcomes	Psychiatric outcomes	Depression	HADs, SADS, BDI, CES-D, SCID, PRIME-MD and SISC	8
		Anxiety	HADs, BAI, SCID, PRIME-MD, SISC and medical records	7
		Psychological distress	HADs and MQOL	2
		Mental status	Medical records	1
		Post-traumatic stress disorder	SCID	1
		Panic disorder	SCID	1
	General outcomes	Pain	SISC and verbal rating scale	3
		Drowsiness	SISC	1
		Nausea	SISC	1
		General malaise	SISC	1
		Weakness	SISC	1
		Breathlessness	SISC	1
Life impact	Spiritual/religious/existential functioning/wellbeing	Desire for death	SADS, YES and SISC	3
		Hopelessness	SADS, SISC and survey question	3
		Being at peace with dying	MMRS and interviews	2
		Sense of burden on others	SPBS	2
		Dissatisfaction with life	SISC	1
		Loss of control	SISC	1
		Loss of dignity	SISC	1
		Loss of interest/pleasure	SISC	1
		Loss of resilience	SISC	1
		Wish to live	YES	1
		Hopefulness	FACT-G	1
		Preparing for death	Survey question	1
		Sense of suffering	SISC	1
		Spiritual crisis	SISC	1
	Emotional functioning/wellbeing	Use of coping strategies/mechanisms	COPE and interviews	3
		Prognostic acceptance	Interviews	1
		Bereavement in caregivers	TRIG	1
		Pre-loss grief in caregivers	PG-13	1
		Having the opportunity to say goodbye to loved ones	Interviews	1
	Cognitive functioning	Cognitive function	MMSE	1
	Social functioning	Communication between patient and family/friends	Medical records and SISC	2
		Social isolation	SISC	1
	Global quality of life	Quality of life	QLQ-C15-PAL, EQ-VAS, SF-36, FACT-G, MQOL, IPOS and interviews	9
	Delivery of care	Treatment preferences (including life-sustaining treatments)	DCS, medical records and interviews	9
		Do-not-resuscitate order completion	Medical records and interviews	5
		End-of-life planning/discussions	Interviews	3
		Dying in hospital	Register data and medical records	2
		Dying in a preferred location	Register data and interviews	2
		Quality of death	GDI and post-mortem survey	2
		Preference for hospice care	Interviews	2
		Preference for comfort care	Register data and interviews	2
		Patient-doctor relationship	THC and PEPPI	1
		Family present at time of death	Register data	1
		Advance directives in place	Medical records	1
		Participation in clinical trials	Interviews	1
		Decisional satisfaction	DCS	1
		Family informed about imminent death	Register data	1
		Bereavement support offered to family	Register data	1
	Perceived health status	Prognostic awareness	Interviews	9
		Prognostic understanding	Interviews and survey question	3
	Personal circumstances	Living will or durable power of attorney in place	Interviews	1
		Financial concerns	SISC	1
Resource use	Hospital/hospice use	Hospice enrolment	Medical records and caregiver report	2
		Length of hospital admission	Register data	1
		Admission to hospital (rate per week)	Register data	1

BAI: Beck Anxiety Inventory; BDI: Beck Depression Inventory; CES-D: Center for Epidemiologic Studies Depression Scale; COPE: Coping Orientation to Problems Experienced Inventory; DCS: Decisional Conflict Scale; EQ-VAS: EuroQol-Visual Analogue Scale; FACT-G: Functional Assessment of Cancer Therapy; GDI: Good Death Inventory; HADs: Hospital Anxiety and Depression Scale; IPOS: Integrated Palliative Care Outcome Scale; ISI: Insomnia Severity Index; MMRS: Multidimensional Measurement of Religiousness/Spirituality for Use in Health Research; MMSE: Mini Mental State Examination; MQOL: McGill Quality of Life Questionnaire; PEPPI: Perceived Efficacy in Patient-Physician Interactions; PG-13: Prolonged Grief Scale; PRIME-MD: Primary Care Evaluation of Mental Disorders; PTSD: Post Traumatic Stress Disorder; QLQ-C15-PAL: Quality of Life Questionnaire Core 15 Palliative Care; SADS: Schedule for Affective Disorders and Schizophrenia; SCID: Structured Clinical Interview for DSM Disorders; SISC: Structured Clinical Interview for Symptoms and Concerns; SPBS: Self-Perceived Burden Scale; THC: The Human Connection Scale; TRIG: The Texas Revised Inventory of Grief; YES: Yale Evaluation of Suicidality.

### Death

The core area of death consisted of one outcome domain: survival.

#### Survival

Four studies reported length of survival, measured in days^27,32,40,44^ most frequently measured using medical records (*n* = 3), apart from one study which used patients’ death certificates to confirm length of survival (*n* = 1).

### Physiological/clinical outcomes

The core area of physiological/clinical outcomes consisted of two outcome domains: psychiatric outcomes and general outcomes.

#### Psychiatric outcomes

The most frequently reported outcomes in this domain were depression and anxiety. Eight studies reported depression, seven in patients^25,33,39,40,44,61,64^ and one in informal caregivers.^42^ In the eight studies, the Hospital Anxiety and Depression Scale (HADS) was the most common measure (*n* = 3). Anxiety was reported in seven studies.^25,28,33,39,40,61,64^ Like depression, anxiety was most frequently measured using HADS (*n* = 3).

Other psychiatric outcomes included psychological distress (feeling depressed, anxious, sad, nervous or worried) (*n* = 2),^25,34^ mental status (*n* = 1),^27^ post-traumatic stress disorder (*n* = 1)^25^ and panic disorder (*n* = 1).^25^

#### General outcomes

The most frequently reported general outcome was pain (*n* = 3 studies).^40,44,64^ This outcome was measured using the Structured Clinical Interview for Symptoms and Concerns (SISC) and numerical rating scales. Other general outcomes were drowsiness (*n* = 1), nausea (*n* = 1), general malaise (*n* = 1), weakness (*n* = 1) and breathlessness (*n* = 1).^64^

### Life impact

This consisted of eight outcome domains: spiritual/religious/existential function/wellbeing, emotional functioning/wellbeing, cognitive functioning, social functioning, global quality of life, delivery of care, perceived health status and personal circumstances.

#### Spiritual/religious/existential functioning/wellbeing

The most frequently reported outcomes for the domain of spiritual/religious/existential functioning/wellbeing were desire for death and hopelessness. Desire for death was reported in three studies,^25,44,64^ measured via the Schedule for Affective Disorders and Schizophrenia (SADS) scale, the Yale Evaluation of Suicidality (YES) and the SISC. Hopelessness was also reported in three studies,^35,44,64^ measured via the SADS scale, the SISC and a survey question. Other outcomes included being at peace with dying (*n* = 2),^25,42^ sense of burden on others (*n* = 2),^51,64^ the wish to live (*n* = 1),^25^ dissatisfaction with life (*n* = 1),^64^ loss of control (*n* = 1),^64^ loss of dignity (*n* = 1),^64^ loss of interest/pleasure (*n* = 1),^64^ loss of resilience (*n* = 1),^64^ hopefulness (*n* = 1),^45^ preparing for death (*n* = 1),^35^ sense of suffering (*n* = 1)^64^ and spiritual crisis (*n* = 1).^64^

#### Emotional functioning/wellbeing

The most frequently reported outcome in this domain was use of coping strategies/mechanisms, reported in three studies^33,39,45^ and measured using the Coping Orientation to Problems Experienced (COPE) inventory and patient interviews.

Other emotional functioning/wellbeing outcomes were prognostic acceptance (*n* = 1),^64^ bereavement in caregivers (*n* = 1),^42^ pre-loss grief in caregivers (*n* = 1)^67^ and having opportunity to say goodbye to loved ones (*n* = 1).^42^

#### Cognitive functioning

Only one study reported cognitive function, defined as any cognitive impairment or decline, as an outcome of prognostication, measured using the Mini Mental State Examination (MMSE).^40^

#### Social functioning

Two studies reported outcomes in this domain, one of which was communication difficulties between patients and family/friends,^28,64^ measured using patients’ medical records and SISC. One study also reported on social isolation as an outcome of prognostication, measured using SISC.^64^

#### Global quality of life

Quality of life was reported in nine studies.^25,26,32,33,39,40,43,51^ MQOL was the most common measure used in four of the studies.

#### Delivery of care

The most frequently reported outcome in this domain was treatment preferences, including preference to receive life-sustaining treatments such as cardiopulmonary resuscitation, intensive care unit, mechanical ventilation, tube feeding, ‘heroic’ measures, chemotherapy and antibiotics. Nine studies reported on patients’ and informal caregivers’ preferences regarding life sustaining treatments post-prognostication.^25,29,34,37,38,47,50,54,65^ This outcome was measured most frequently via interviews with patients or informal caregivers (*n* = 5), followed by medical record reviews (*n* = 4). Similarly, do-not-resuscitate order completion was reported in five studies,^25,46,47,49,65^ measured via interviews with patients or informal caregivers (*n* = 3) and patients’ medical records (*n* = 2). End-of-life planning/discussions were reported as outcomes of prognostication in three studies,^25,47,52^ ascertained from interviews with patients or informal caregivers.

Other outcomes of delivery of care were patient-doctor relationship (*n* = 1),^30^ dying in hospital (*n* = 2),^24,38^ dying in a preferred location (*n* = 2),^38,67^ quality of death (*n* = 2),^25,27^ preference for hospice care (*n* = 2),^34,36^ preference for comfort care (*n* = 2),^34,48^ having family present at time of death (*n* = 1),^48^ having advance directives in place (*n* = 1),^65^ participation in clinical trials (*n* = 1),^47^ decisional satisfaction (*n* = 1),^37^ family being informed about patient’s imminent death (*n* = 1)^48^ and bereavement support being offered to family (*n* = 1).^48^

#### Perceived health status

Prognostic awareness, defined as the awareness of shortened life expectancy, was reported as an outcome in nine studies.^25,26,32,43,45–47,51,54^ All studies measured this by interviewing patients. Prognostic understanding refers to the perception of prognosis and was reported in three studies (*n* = 3) measured by interviews and survey questionnaires.^34,39,49^

#### Personal circumstances

These were defined as outcomes relating to patients’ finances, home and environment.^21^ One study (*n* = 1) ascertained whether knowing their prognosis affected if patients had a living will or durable power of attorney in place (measured through interviews).^25^ Another study (*n* = 1) reported the outcome of financial concerns,^64^ measured via the SISC.

### Resource use

This area consisted of one outcome domain: hospital/hospice use.

#### Hospital/hospice use

The most frequently reported outcome of this domain was hospice enrolment, reported by two studies (*n* = 2).^31,38^ This outcome was measured via informal caregiver reports and patients’ medical records.

One study (*n* = 1) reported admission to hospital (rate per week) as an outcome, as well as length of hospital admission (in days).^24^ Both outcomes were ascertained from national register data.

### Results of qualitative synthesis

Nine studies provided qualitative evidence on patients’ and informal caregivers’ experiences and perceptions of prognostication in advanced cancer.^55–63^ Across the qualitative data, 23 outcomes of prognostication were identified. Table 4 shows the themes identified in the qualitative data. Additional quotations are presented in Supplementary Appendix 4.

**Table 4.** Themes identified in the qualitative data, according to the core areas and domains within the COMET taxonomy.

Core area	Domain	Theme	No. of studies reporting data on this theme (*n* = 9)
Physiological/clinical	Psychiatric outcomes	Psychological status	1
Life impact	Spiritual/religious/existential functioning/wellbeing	Maintaining hope	4
		Preparedness for end-of-life	4
		Loss of hope	1
		Worry about dying	1
	Emotional functioning/wellbeing	Avoidance/denial	6
		Caregiver regret	1
		Emotional distress	1
		Frustration	1
		Having the opportunity to say goodbye to loved ones	1
	Social functioning	Communication between patient and family/friends	1
		Patient-caregiver relationship	1
	Global quality of life	Quality of life	1
	Delivery of care	Treatment preferences	4
		Conflicting preferences for prognostic information between patients and caregivers	3
		Having a survival timeframe	3
		Needing additional information	3
		Change in information needs/preferences	2
		Patient-doctor relationship	1
	Perceived Health Status	Being aware of prognostic uncertainty	3
		Prognostic understanding	2
		Prognostic awareness	1
	Personal circumstances	Getting affairs in order	3

### Physiological/clinical outcomes

#### Psychiatric outcomes

One study described patients’ and informal caregivers’ experiences of prognostication in association with psychological status.^58^ Not knowing their prognosis evoked uncertainty in some patients, which in turn affected their psychological state. However, awareness of prognosis was also noted to affect patients psychologically:

He was aware of his prognosis. But during the final week he slipped into depression suddenly and the final two days were the worst (Informal caregiver, page 115).^58^

### Life impact

#### Spiritual/religious/existential functioning/wellbeing

Seven studies identified spiritual/religious/existential functioning/wellbeing experiences associated with prognostication. Themes were developed in relation to maintaining hope,^55,56,60,62^ preparedness for end-of-life,^55,57,58,60^ loss of hope,^56^ control,^56^ and worry about dying.^61^

It was clear from narratives that many patients and informal caregivers, while aware of the limited life expectancy, had a continuing need for hope. This need was intertwined with their experience of prognostic disclosure, where it was generally felt that the mode and manner of communication of prognosis should maintain hope. For example, one patient described their desire for ‘a little bit of hope’, regardless of their prognosis.^56^ Conversely, others avoided specific prognostic discussions in order to preserve hope:

I don’t know how much time (the patient) has left. We always approach things, every therapy, with the notion of hope, that it will either control or hopefully cure his condition. (Informal caregiver, page 820).^62^

Linked to this, one study highlighted how prognostic information could also produce loss of hope in patients:

[The doctor] is not God so he can’t say exactly you have six months. I think he gave away hope. In dad’s eyes I can see that he lost a bit of hope. (Informal caregiver, page 5).^56^

Study findings were mixed on how prognostication affected patients’ and informal caregivers’ preparedness for end-of-life.^55,57,58,60^ In general, patients and informal caregivers were prepared for end-of-life because they were fully informed of their prognosis, whilst a lack of prognostic information impacted negatively on informal caregivers’ preparedness, such as funeral arrangements.

Finally, prognostication evoked worries about dying in some patients, with concerns about ‘what will happen at the end,’ such as when and how they might die and who might find them.^61^

#### Emotional functioning/wellbeing

Six studies discussed this theme in relation to experiences of avoidance/denial,^55,56,60–63^ caregiver regret,^60^ emotional distress,^56^ frustration,^61^ and having the opportunity to say goodbye to loved ones.^60^

Prognostication had a profound effect on patients and informal caregivers who had to come to terms with limited life expectancy, which could produce emotional distress^56^ and frustration.^61^ Information around prognosis evoked frustration in some patients, with one, in particular, stating that they had *‘*still lots of things’ they wanted to achieve.^61^ Similarly, some prognostic information, particularly about palliative care referral, caused emotional distress:

And she said I have come to talk to you about palliative care, and he just went into an absolute heap. And of course, that word when you say ‘palliative care’ he immediately thought death in three months. He just went into absolute shock—burst into tears. . . It was too soon. (Informal caregiver, page 5).^56^

Patients and informal caregivers mitigated the emotional impact of prognostication by avoiding information about their prognosis or denying the implications of the information they received.^55,56,60–63^ Reasons for avoidance included being ‘too scared to ask’^61^ and to preserve hope.

Avoidance was not always preferable, however, and some informal caregivers wished that they had received prognostic information sooner. Others misunderstood prognostic information, leading to regrets. Some informal caregivers voiced regrets for not using their remaining time with their loved ones better due to not receiving prognostic information earlier:

Maybe we could have changed our discussions from the fight and the forward thinking of what we’ll do next, to what needed to be said to each other right then, right when our last few conversations meant the most. (Informal caregiver, page 1474).^60^

Similarly, one informal caregiver expressed a desire to have known their loved one's prognosis sooner so that they could have had the opportunity to say goodbye.^60^

#### Social functioning

Two studies observed an association between prognostication and social functioning.^56, 57^ The patient-caregiver relationship was affected by not discussing impending death:

We haven’t talked about death, for example. . .And not having those kind [sic] of talks has affected our relationship. . . (Informal caregiver, page 1415).^57^

There was also evidence that communication changed between patients and informal caregivers, who, after receiving the prognosis, no longer communicated openly with each other.^56^

#### Global quality of life

One study highlighted the benefit for patients’ quality of life of having an indication of potentially increased life expectancy rather than no prognosis at all:

Her oncologist said to her, ‘I want to continue with the treatment, there’s a 30% chance here.’ He has not ever said a 30% chance of what or for how long, but just hearing that has been what has kept her quality of life for these past six months so much more bearable and better than without hearing that. (Informal caregiver, page 5).^56^

#### Delivery of care

Seven studies noted that prognostication had an impact on patient-doctor relationships,^56^ treatment preferences,^56,59,60,63^ change in information needs/preferences,^55,56^ conflicting preference for prognostic information between patients and caregivers,^56,57,62^ having a survival timeframe,^56,59,60^ and needing additional information.^56,60,62^

Prognostication and the extent of its communication affected patient-doctor relationships. Some patients felt supported by clinicians who disclosed their prognosis, whilst others experienced feelings of abandonment and betrayal after their doctor did not contact them again after this disclosure.^56^

Prognostication also influenced treatment preferences of patients and informal caregivers.^56,59,60,63^ Informal caregivers identified the need for a timeframe for planning care.^62^ Some described how prognostication provided an opportunity to explore alternative treatments, such as herbal therapies,^56^ whilst others felt they would not have continued with cancer treatment if they had a better understanding of the patient’s prognosis:

Well, none of us would have made the decisions we did [to continue treatment] if we had known the truth about her illness. I just don’t know if the doctors knew, but they must have. Why wouldn’t they tell us? You have got to wonder why they put her through all that—I mean the chemo and especially the radiology and all those burns. She was in pain and had burns everywhere from the radiation. It was awful. She wouldn’t have gone through it if she had known what they knew, but they told us it was curable; so what are you going to do? (Informal caregiver, page 1182).^63^

Information preferences of patients and informal caregivers were variable and prone to change.^55,56^ Many patients decided that they no longer wanted updates regarding their prognosis,^56^ whilst some patients and informal caregivers had conflicting preferences for prognostic information.^56,57,62^

Information preferences of patients and informal caregivers included being given a survival timeframe.^56,59,60^ Both positive and negative aspects of this were identified; some felt that it allowed for future planning and facilitated saying goodbye to loved ones.^59,60^ Study respondents in favour said that they could be told the average survival time of patients with their illness or even just a rough range.^59^ On the other hand, some felt that having a timeframe could make it difficult for patients to come to terms with their limited survival,^56^ and even cause distress:

The danger is that if you put a time frame on it, that person will believe you. . .the closer it gets the more freaked out they get. That happened to me wife, that is the median, she believed it and was almost counting the weeks away. (Informal caregiver, page 737).^59^

Some patients and informal caregivers stated that they needed additional information about their prognosis, which they obtained either from their clinicians^60^ or from secondary sources to supplement what clinicians had told them.^56,62^

#### Perceived health status

Three linked themes were developed in relation to perceived health status, including prognostic awareness,^58^ prognostic understanding,^56,60^ and being aware of prognostic uncertainty.^57,59,60^

Prognostic awareness was something that patients could neither escape nor ignore. Often, patients who were not initially aware of their prognosis were said to become aware, or at least suspect it, due to the progression of their disease and decline in physical state:

He was aware of his diagnosis, but he did not know of the prognosis. He was very much worried about his condition and started fearing that something bad was going to happen. (Informal caregiver, page 115).^58^

On the other hand, prognostic understanding was something that had to be sought by patients and informal caregivers or encouraged by clinicians.^56,60^ It was viewed to be clinicians’ responsibility to nurture prognostic understanding in patients and informal caregivers so that they might make decisions accordingly.

Regardless of whether patients and informal caregivers had awareness or understanding of their prognosis, they were unanimous that clinicians should be honest about the level of uncertainty that comes with making an estimate of life expectancy.^57^ Some patients acknowledged the uncertainty of prognostication and sympathised with clinicians who had to provide such estimates.^59^ Other studies found that informal caregivers considered that clinicians were not always open about prognostic uncertainty:

We were never given any indication that my wife was going to die. . .Only after her death, when I questioned him did he acknowledge that his prognosis of a cure had changed and he was just hoping to put her into remission (Informal caregiver, page 1473).^60^

#### Personal circumstances

Prognostication impacted the way in which patients and informal caregivers handled their personal circumstances.^56,58,59^ Discussing prognosis allowed individuals to settle their responsibilities, plan for the future and get their affairs in order:

As I said no-one’s god and no-one can say your time’s going to be up in 6 months, but I think if you’ve got some idea. . .you can put your life in order and get your family and that prepare a but, I think that’s good. (Patient, page 737).^59^

### Combined results

Outcomes with similar definitions were merged in order to combine outcomes identified in both types of studies into a single list. For example, psychological status and mental status were merged as psychological/mental status. This resulted in a final list of 70 outcomes of prognostication in advanced cancer (Table 5).

**Table 5.** List of all outcomes identified by the systematic review, according to the core areas and domains within the COMET taxonomy.

Core Area	Domain	Outcome	No. of studies reporting data on this outcome (*n* = 42)
Death	Survival	Length of survival (days)	4
Physiological/clinical outcomes	Psychiatric outcomes	Depression	8
		Anxiety	7
		Psychological/mental status	2
		Psychological distress	2
		Post-traumatic stress disorder	1
		Panic disorder	1
	General outcomes	Pain	3
		Drowsiness	1
		Nausea	1
		General malaise	1
		Weakness	1
		Breathlessness	1
Life impact	Spiritual/religious/existential functioning/wellbeing	Hopefulness/maintaining hope	5
		Preparedness for end-of-life	5
		Hopelessness/loss of hope	4
		Desire for death	3
		Being at peace with dying	2
		Perceived sense of burden on others	2
		Dissatisfaction with life	1
		Loss of control	1
		Loss of dignity	1
		Loss of interest/pleasure	1
		Loss of resilience	1
		Wish to live	1
		Worry about dying	1
		Sense of suffering	1
		Spiritual crisis	1
	Emotional functioning/wellbeing	Avoidance/denial	6
		Use of coping strategies/mechanisms	3
		Having the opportunity to say goodbye to loved ones	2
		Caregiver regret	1
		Emotional distress	1
		Frustration	1
		Prognostic acceptance	1
		Bereavement in caregivers	1
		Pre-loss grief in caregivers	1
	Cognitive functioning	Cognitive function	1
	Social functioning	Communication between patient and family/friends	3
		Patient-caregiver relationship	1
		Social isolation	1
	Global quality of life	Quality of life	10
	Delivery of care	Treatment preferences (including life-sustaining treatments)	13
		Do-not-resuscitate order completion	5
		Conflicting preferences for prognostic information between patients and caregivers	3
		Having a survival timeframe	3
		Needing additional information	3
		End-of-life planning/discussions	3
		Change in information needs/preferences	2
		Dying in hospital	2
		Dying in a preferred location	2
		Quality of death	2
		Preference for hospice care	2
		Preference for comfort care	2
		Patient-doctor relationship	2
		Family present at time of death	1
		Advance directives in place	1
		Participation in clinical trials	1
		Decisional satisfaction	1
		Family informed about imminent death	1
		Bereavement support offered to family	1
	Perceived health status	Prognostic awareness	10
		Prognostic understanding	5
		Being aware of prognostic uncertainty	3
	Personal circumstances	Getting affairs in order	3
		Living will or durable power of attorney in place	1
		Financial concerns	1
Resource use	Hospital/hospice use	Hospice enrolment	2
		Length of hospital admission	1
		Admission to hospital (rate per week)	1

## Discussion

### Main findings

We identified 70 outcomes used to measure the impact of prognostication in 42 included studies pertaining to 12 of the COMET taxonomy domains. The most prevalent outcomes were treatment preferences (*n* = 13), prognostic awareness (*n* = 10), quality of life (*n* = 10) and depression (*n* = 8). Prognostication was shown to have a widespread impact on patients and informal caregivers, with the highest number of outcomes categorised under the delivery of care domain (*n* = 50), followed by spiritual/religious/existential functioning/wellbeing (*n* = 30) and psychiatric outcomes (*n* = 21).

Half of the outcomes (*n* = 35) were identified in only one study each, and no outcome was reported by all studies, reflecting the diversity of outcomes chosen. This systematic review thus demonstrates the variation of outcomes used for assessing impact in prognostic studies in advanced cancer. The results of the quantitative analysis, in particular, highlight the heterogeneity and inconsistencies in the outcomes included in studies and how they are assessed. Some studies used non-validated methods, such as unstructured patient or caregiver reports, for assessing outcomes, risking reporting and recall bias.^68^ Studies that used validated outcome measures varied widely in the measures chosen. Seven different measures were used to assess depression and six to assess quality of life, each with varying quality and validity for use in palliative care.^69,70^ Evaluation of specific measures was outside the scope of this review; further research is needed to evaluate the suitability of outcome measures used in prognostic studies within an advanced cancer population.

The patient perspective is increasingly understood as important for assessing and identifying healthcare outcomes.^71^ By including qualitative data on the personal experiences of patients and informal carers, we identified key outcomes of prognostication that are meaningful to people receiving care that quantitative studies did not identify. The discordance between the number of qualitative and quantitative papers identified in our search indicates a dearth of literature on lived experiences of prognostication in advanced cancer. Patients’ and informal caregivers' experiences of prognostication can provide insights about care preferences and how these align with or differ from current clinical practice.^71^ There is, therefore, a need for further consideration of which outcomes of prognostication are deemed important to patients and informal caregivers to capture these outcomes in future prognostic research.

### What this study adds?

An important component of study design is choosing suitable outcome measures. It can be difficult to compare study findings across specific areas of research when endpoints are incompatible, reducing the potential for meta-analyses and perpetuating outcome reporting biases.^72^ Prognostic studies have often focused on outcomes deemed important by academics and clinicians, and outcomes used in some studies may not be meaningful for patients or informal caregivers.^72,73^ Studies should always account for the opinions and experiences of patients and other personally affected stakeholders, not least in order to facilitate the translation of findings into clinical practice.^74–77^ Our systematic review identified considerable heterogeneity in outcome reporting across included studies and under-representation of patients’ and informal caregivers’ experiences. These findings could now be used to inform the development of a core outcome set for prognostication in advanced cancer in consultation with relevant stakeholders.

### Strengths and limitations

This is the first systematic review to synthesise data on outcomes from both quantitative and qualitative studies of prognostication in advanced cancer reported in English. An extensive search ensured a transparent, replicable report. Rigorous screening identified all relevant studies, so this review synthesises all available evidence written in English.

This review has some limitations. The research questions and analysis only included adult participants; therefore, results are not generalisable to a paediatric population. We only included publications written in English. However, international publications were included, reducing the likelihood of selection bias.^78^

Six of the non-randomised studies were all conducted by the same authors in homogenous populations.^34,43,50–53^ These studies were conducted in different years, but we could not determine whether the same individuals had been enrolled in more than one of these studies, which might have biased effect sizes.^79^ However, since the aim of our review was to narratively synthesise the outcomes of prognostication rather than investigate effect sizes, this does not affect our findings.

Prognostic interventions used in the included studies were not discussed in this review, due to heterogeneity or inadequate descriptions of the methods of prognostication used. Future exploration of interventions used in prognostic studies may allow for the interpretation of our findings regarding the relationship between prognostic interventions and outcomes of prognostication.

We were unable to distinguish between outcomes due solely to prognostication and outcomes arising from the underlying disease, such as pain or fatigue, and our review did not seek to make such distinctions. However, there is likely to be some confounding when assessing the relationship between prognostication and these kinds of outcomes, which further supports the need for a core outcome set for prognostication in advanced cancer.

Finally, there is currently no universally accepted method of classifying outcomes into domains. The COMET taxonomy^21^ provided useful guidance about how to systematically group outcomes but posed some challenges. The COMET guidance on how to allocate outcomes to a specific domain was sometimes ambiguous, and some domains lacked suitable sub-categories for some outcomes, particularly those relating to spiritual, religious and existential functioning/wellbeing. The taxonomy developers permit and encourage further development of sub-categories to provide finer classification within each of the outcome domains.^21^ Duplication or overlap of outcomes or domains does not lead to loss or misclassification of information when developing a core outcome set. We, therefore, created our own domain and sub-categories for this particular group of outcomes.

### Conclusion

This review demonstrates that studies of prognostication in advanced cancer vary widely in how they report and measure outcomes. In addition, experiential outcomes for patients and informal caregivers are not always represented. In order to conduct future research into the impact of prognostication, a standardised approach to outcome reporting in prognostic studies is required. This should be done in consultation with key stakeholders to ensure outcomes reported are relevant and meaningful to those the research affects the most.

## Supplemental Material

sj-pdf-1-pmj-10.1177_02692163231191148 – Supplemental material for What outcomes do studies use to measure the impact of prognostication on people with advanced cancer? Findings from a systematic review of quantitative and qualitative studies Click here for additional data file. Supplemental material, sj-pdf-1-pmj-10.1177_02692163231191148 for What outcomes do studies use to measure the impact of prognostication on people with advanced cancer? Findings from a systematic review of quantitative and qualitative studies by Caitlin Spooner, Bella Vivat, Nicola White, Andrea Bruun, Gudrun Rohde, Pei Xing Kwek and Patrick Stone in Palliative Medicine

sj-pdf-2-pmj-10.1177_02692163231191148 – Supplemental material for What outcomes do studies use to measure the impact of prognostication on people with advanced cancer? Findings from a systematic review of quantitative and qualitative studies Click here for additional data file. Supplemental material, sj-pdf-2-pmj-10.1177_02692163231191148 for What outcomes do studies use to measure the impact of prognostication on people with advanced cancer? Findings from a systematic review of quantitative and qualitative studies by Caitlin Spooner, Bella Vivat, Nicola White, Andrea Bruun, Gudrun Rohde, Pei Xing Kwek and Patrick Stone in Palliative Medicine

sj-pdf-3-pmj-10.1177_02692163231191148 – Supplemental material for What outcomes do studies use to measure the impact of prognostication on people with advanced cancer? Findings from a systematic review of quantitative and qualitative studies Click here for additional data file. Supplemental material, sj-pdf-3-pmj-10.1177_02692163231191148 for What outcomes do studies use to measure the impact of prognostication on people with advanced cancer? Findings from a systematic review of quantitative and qualitative studies by Caitlin Spooner, Bella Vivat, Nicola White, Andrea Bruun, Gudrun Rohde, Pei Xing Kwek and Patrick Stone in Palliative Medicine

sj-pdf-4-pmj-10.1177_02692163231191148 – Supplemental material for What outcomes do studies use to measure the impact of prognostication on people with advanced cancer? Findings from a systematic review of quantitative and qualitative studies Click here for additional data file. Supplemental material, sj-pdf-4-pmj-10.1177_02692163231191148 for What outcomes do studies use to measure the impact of prognostication on people with advanced cancer? Findings from a systematic review of quantitative and qualitative studies by Caitlin Spooner, Bella Vivat, Nicola White, Andrea Bruun, Gudrun Rohde, Pei Xing Kwek and Patrick Stone in Palliative Medicine
