# Supplementary material for: What outcomes do studies use to measure the impact of prognostication on people with advanced cancer? Findings from a systematic review of quantitative and qualitative studies
Source: Palliat Med. 2023 Aug 10;37(9):1345–64. doi: 10.1177/02692163231191148 (PMC10548779; doi:10.1177/02692163231191148)
Supplement: sj-pdf-1-pmj-10.1177_02692163231191148 – Supplemental material for What outcomes do studies use to measure the impact of prognostication on people with advanced cancer? Findings from a systematic review of quantitative and qualitative studies [file sj-pdf-1-pmj-10.1177_02692163231191148.pdf]

Supplemental Appendix 1: Search strategy

This strategy was employed on MEDLINE and modified for other databases

1. exp Palliative Care/
2. palliative care.tw.
3. exp Terminal Care/
4. (terminal* adj2 care).tw.
5. Hospices/
6. hospice care.tw.
7. end stage.tw.
8. late stage.tw.
9. (advanced adj2 disease*).tw.
10. (advanced adj2 illness*).tw.
11. terminal* ill*.tw.
12. end of life.tw.
13. end-of-life.tw.
14. **OR 1-13**

15. exp Neoplasms/
16. neoplas*.tw.
17. oncolog*.tw.
18. cancer*.tw.
19. carcinoma*.tw.
20. tumor*.tw.
21. tumour*.tw.
22. sarcoma*.tw.
23. malignan*.tw.
24. metasta*.tw.
25. **OR 15-24**

26. prognostication.tw.
27. prognos* estimat*.tw.
28. prognos* predict*.tw.
29. surviv* estimat*.tw.
30. surviv* predict*.tw.
31. mortality estimat.tw.
32. mortality predict*.tw.
33. exp Life Expectancy/
34. (estim* adj2 surviv*).tw.
35. (prognos* adj3 (scor* or tool* or model* or algorithm* or index* or scale*)).tw.
36. Decision Making/
37. (prognos* or mortality or "life expectancy" or survival or disease course) adj2 (discuss* or conversat* or disclos* or communicat* or consult* or understand* or aware* or inform*).tw.
38. **OR 26-37**

39. outcome.tw.
40. effect*.tw.
41. impact*.tw.
42. benefit*.tw.
43. item*.tw.
44. factor*.tw.
45. issue*.tw.

46. compar*.tw.
47. OR 39-46

48. 14 AND 25 AND 38 AND 47

49. Limit 48 to (English language and "all adult (18 plus years)" and "humans only (removes records about animals)")
