# Supplementary material for: What outcomes do studies use to measure the impact of prognostication on people with advanced cancer? Findings from a systematic review of quantitative and qualitative studies
Source: Palliat Med. 2023 Aug 10;37(9):1345–64. doi: 10.1177/02692163231191148 (PMC10548779; doi:10.1177/02692163231191148)
Supplement: sj-pdf-2-pmj-10.1177_02692163231191148 – Supplemental material for What outcomes do studies use to measure the impact of prognostication on people with advanced cancer? Findings from a systematic review of quantitative and qualitative studies [file sj-pdf-2-pmj-10.1177_02692163231191148.pdf]

Supplemental Appendix 2: Data extraction form

1. General information	
Date form completed *(dd/mm/yyyy)*	
Name of extractor	
Title of paper	
Authors of paper	
Year of publication	

2. Methodology	
Study design *(e.g., quantitative, qualitative or mixed methods)*	
Methodology *(e.g., cohort, case-control, ethnography etc.)*	
Number of participants	
Study duration	
Participant population	
Geographical setting *(e.g., country)*	
Study setting *(e.g., inpatient, outpatient, community, hospice)*	
Data collection methods *(e.g., survey, registry, medical record review, interview, etc.)*	
**Notes:**	

FOR QUANTITATIVE STUDIES (delete as appropriate)

COMET taxonomy guidance available at: https://www.comet-initiative.org/assets/downloads/Taxonomy%20explanation%20table%202018.10.30.pdf

3. Outcomes			
Patient outcome	Assessment	COMET taxonomy core area	COMET taxonomy outcome domain
Results			
**Notes:**			

FOR QUALITATIVE STUDIES (delete as appropriate)

3. Outcomes		
Themes or Subtheme	Result	Illustration (a direct quote from a participant, an observation, or other supporting data from the paper)

Key conclusions of study authors	
Reviewer's comments	
**Notes:**	
