# Supplementary material for: What outcomes do studies use to measure the impact of prognostication on people with advanced cancer? Findings from a systematic review of quantitative and qualitative studies
Source: Palliat Med. 2023 Aug 10;37(9):1345–64. doi: 10.1177/02692163231191148 (PMC10548779; doi:10.1177/02692163231191148)
Supplement: sj-pdf-3-pmj-10.1177_02692163231191148 – Supplemental material for What outcomes do studies use to measure the impact of prognostication on people with advanced cancer? Findings from a systematic review of quantitative and qualitative studies [file sj-pdf-3-pmj-10.1177_02692163231191148.pdf]

Supplemental Appendix 3: Quality assessment of included studies using the MMAT

	QUALITATIVE					RANDOMISED CONTROLLED TRIALS					NON-RANDOMISED QUANTITATIVE STUDIES					MIXED METHODS					OVERALL QUALITY SCORE
	Is the qualitative approach appropriate to answer the research question?	Are the qualitative data collection methods adequate to address the research question?	Are the findings adequately derived from the data?	Is the interpretation of results sufficiently substantiated by data?	Is there coherence between qualitative data sources, collection, analysis and interpretation?	Is randomization appropriately performed?	Are the groups comparable at baseline?	Are there complete outcome data?	Are outcome assessors blinded to the intervention provided?	Did the participants adhere to the assigned intervention?	Are the participants representative of the target population?	Are measurements appropriate regarding both the outcome and intervention (or exposure)?	Are there complete outcome data?	Are the confounders accounted for in the design and analysis?	During the study period, is the intervention administered (or exposure occurred) as intended?	Is there an adequate rationale for using a mixed methods design to address the research question?	Are the different components of the study effectively integrated to answer the research question?	Are the outputs of the integration of qualitative and quantitative components adequately interpreted?	Are divergences and inconsistencies between quantitative and qualitative results adequately addressed?	Do the different components of the study adhere to the quality criteria of each tradition of the methods?	4-5 = High quality<br>3 = Moderate quality<br>0-2 = Low quality
Aabom et al. 2005											YES	YES	YES	YES	YES						5
Ahn et al. 2013											YES	YES	NO	YES	YES						4
An et al. 2014											YES	CAN'T TELL	NO	YES	YES						3
Applebaum et al.	CAN'T TELL	YES	YES	YES	YES						NO	CAN'T TELL	CAN'T TELL	NO	YES	NO	YES	YES	NO	NO	2
Baek et al. 2012											YES	CAN'T TELL	NO	YES	YES						3
Barnett 2006	CAN'T TELL	YES	NO	NO	NO						YES	YES	YES	NO	YES	NO	YES	YES	YES	NO	3
Bradely et al. 2001											NO	YES	NO	YES	YES						3
Chan 2011											YES	YES	CAN'T TELL	YES	YES						4
Chen et al. 2019						CAN'T TELL	YES	YES	YES	YES											4
Cherlin et al. 2005	YES	YES	YES	NO	YES						NO	NO	YES	NO	CAN'T TELL	YES	NO	NO	NO	NO	2
Chochinov et al. 2000											NO	YES	CAN'T TELL	YES	CAN'T TELL						2
Clayton et al. 2005	CAN'T TELL	YES	CAN'T TELL	YES	YES																3

Fenton et al. 2018											YES	YES	CAN'T TELL	YES	YES						4
Friedrichsen et al. 2011	CAN'T TELL	YES	YES	YES	YES																4
Gramling et al. 2019a											NO	YES	NO	YES	YES						3
Gramling et al. 2019b											NO	YES	YES	YES	YES						4
Helft et al. 2003											NO	NO	NO	YES	YES						2
Janssens et al. 2019											NO	YES	YES	CAN'T TELL	YES						3
Kao et al. 2014											NO	YES	NO	CAN'T TELL	NO						1
Kim et al. 2013											NO	YES	YES	YES	YES						4
Kirk et al. 2004	CAN'T TELL	YES	YES	YES	YES																4
Lambden et al. 2016											YES	NO	NO	CAN'T TELL	NO						1
Lee et al. 2020											NO	YES	CAN'T TELL	YES	YES						3
Lundquist et al. 2011											YES	CAN'T TELL	YES	CAN'T TELL	NO						2
Nielsen et al. 2017											YES	NO	NO	YES	YES						3
Nipp et al. 2017											YES	YES	YES	NO	YES						4
Park et al. 2015	NO	NO	YES	YES	YES																3
Park et al. 2016											NO	YES	CAN'T TELL	YES	YES						3
Ray et al. 2006											YES	YES	YES	YES	YES						5
Røen et al. 2018	YES	YES	YES	YES	YES																5
Sudhakar et al. 2021	YES	YES	YES	CAN'T TELL	YES																4
Shen et al. 2018											CAN'T TELL	YES	CAN'T TELL	YES	NO						2
Tang et al. 2016a											NO	CAN'T TELL	NO	YES	NO						1
Tang et al. 2016b											NO	YES	NO	YES	NO						2
Tang et al. 2018											NO	NO	NO	YES	NO						1
Tang et al. 2019											NO	YES	NO	YES	NO						2
Tang et al. 2008											NO	YES	NO	YES	YES						3
Tang et al. 2014											NO	YES	YES	YES	YES						4

Thompson et al. 2009											YES	YES	NO	CAN'T TELL	YES	NO	NO	YES	NO	NO	2
Vlckova et al. 2022											YES	YES	YES	YES	YES						5
Wen et al. 2019											NO	CAN'T TELL	NO	YES	YES						2
Yoshida et al. 2011											YES	YES	NO	YES	YES						4
