# Supplementary material for: What outcomes do studies use to measure the impact of prognostication on people with advanced cancer? Findings from a systematic review of quantitative and qualitative studies
Source: Palliat Med. 2023 Aug 10;37(9):1345–64. doi: 10.1177/02692163231191148 (PMC10548779; doi:10.1177/02692163231191148)
Supplement: sj-pdf-4-pmj-10.1177_02692163231191148 – Supplemental material for What outcomes do studies use to measure the impact of prognostication on people with advanced cancer? Findings from a systematic review of quantitative and qualitative studies [file sj-pdf-4-pmj-10.1177_02692163231191148.pdf]

Supplemental Appendix 4: Additional quotations

Core Area	Domain	Theme	No. of studies reporting on this theme (*n* = 9)	Illustration (a direct quote from a participant, an observation, or other supporting data from the paper)	Authors interpretation
Physiological/clinical	Psychiatric outcomes	Psychological status	1	"He was aware of his prognosis. But during the final week he slipped into depression suddenly and the final two days were the worst" (Informal Caregiver; Sudhakar et al, page 115)	The knowledge of prognosis was found to cause major effect in the psychological state and preparedness of the patient. (Sudhakar et al, page 114).
				"He was aware of his diagnosis, but he did not know his prognosis. He was very much worried about his condition and started fearing that something bad was going to happen." (Informal caregiver; Sudhakar et al, page 115)	Similar to the awareness of prognosis, being unaware also yielded similar psychological responses from the patients, while majority of the caregivers believed that unawareness among the patients led to uncertainty, which in turn had worsened their psychological health during the final week. (Sudhakar, page 115).
Life impact	Spiritual/religious/ existential functioning/wellbeing	Maintaining hope	4	"We're hopeful people and we need to believe that there's hope, and we're optimistic people and we need to progress with what we've got." (Informal caregiver; Applebaum et al, page 820).	Many informal caregivers wanted to maintain their - or the patient's - hope for the future. Specifically, for several informal caregivers, having open conversations about prognosis was perceived as discordant with maintaining hope. Others reported allowing prognostic awareness and hope to co-exist. (Applebaum et al, page 821).
				"We've preferred to keep optimism as the front and center, with a lot of prayers backing it up." (Informal caregiver; Appelbaum et al, page 820).	
				"I don't know how much time (the patient) has left. We always approach things, every therapy, with the notion of hope, that it will either control or hopefully cure his condition." (Informal caregiver; Applebaum et al, page 820).	
				"We know it's bad and we're really focusing on treating it rather than worrying about it…He's got a great attitude. And we're basically taking it day	

				by day." (Informal caregiver; Applebaum et al, page 820).	
				"You should not know more than you can take, so to say. Otherwise you can be really frightened and depressed. // physicians knows exactly, they have seen the last phase of life. I don't think it's necessary for them to tell patients every detail about the future. You can five some doses now and again." (Patient; Friedrichsen et al, page 177).	Some patients wanted to hear partial truths to maintain some hope. (Friedrichsen et al, page. 177).
				"I don't want to hear that I have one year left. I want him (the physician) to say, "it didn't go to as we planned, but we continue anyway (with treatment)". Then you still have the carrot in front of you. I want him to say that I still have 10-12 year, that's what I really want". (Patient; Friedrichsen et al, page 176).	Some patients made a distinction between the truth that was provided by their physician and what they wanted to hear, their inner desirable truth…this kind of truth was always present in their inner reality and gave them home, even if they know that they were dying. (Friedrichsen et al. 2011, page 176).
				"I don't want to know about [palliative care] at the moment. I sort of live in hope and the chemotherapy has done wonders for her…there is probably a lot they could do for my mother but I sort of associate palliative care with the dying process...That's why I get upset to even think about it." (Informal caregiver; Kirk et al, page 4).	Patients expressed a continuing need for hope even when they knew and accepted that they were in the terminal stages of disease and had a limited life expectancy. Even in the end stages, patients and families still wanted the door to be left open for the possibility of a miracle; many expressed a compartmentalised awareness, simultaneously acknowledging the terminal nature of the illness, while retaining a need and sense of hope. (Kirk et al. 2004, page 4).
				"Well, I feel invincible, even though I know it’s growing again I still feel invincible but I still know that I’m going to die ...I manage to have these two things in my head at the same time, right and left." (Patient; Kirk et al, page 5).	
				"He knows the extent of his illness, but when he’s talking to me, [it’s as if] he’s going to be here forever." (Informal caregiver; Kirk et al, page 5).	

				"We want the information but there has to be a sliver of hope left…Her oncologist said to her, 'I want to continue with the treatment, there's a 30% chance of what or for here.' He has not ever said a 30% chance of what or for how long, but just hearing that has been what has kept her quality of life for these past six months so much more bearable and better than without hearing that " (Informal caregiver; Kirk et al, page 5).	
				"I don't mind what I hear, so long as there is a little light at the end of the tunnel you know what I mean? ...a little bit of hope there, yes." (Patient; Kirk et al, page 5)	
				"I would like to know the truth, but there is a way between saying 'Well, you have cancer of the cervix and it's incurable,' instead of, 'We'll do our best . . . and there are miracles in the world' [a miracle] still can happen . . . I'm still here [laughs]." (Patient; Kirk et al, page 5).	
				"Sooner - finding that balance between hope/optimism and realism, by the time it spread to her brain it was too late." (Informal caregiver; Park et al. 2015, page 1473).	Often, men believed that the physician, their wife, or thy themselves had avoided these conversations in order to preserve hope. (Park et al. 2015, page 1473).
				"Her doctors always tried to stay positive but never truly came out and said she was going to die. They repeatedly told us they'd know when we got to that point. That talk never really came. We stayed upbeat and positive until the end. In retrospect, I'm still unsure if knowing for sure or holding strong hope would be better." (Informal caregiver; Park et al. 2015, page 1474).	Several respondents recognized the difficulty in balancing clear and honest communication with the need for hope, many acknowledging their own ambivalence about which they preferred. (Park et al. 2015, page 1474).

		Preparedness for end-of-life	4	"No, there's not limit. Because...let's say that they tumour will be growing fact. Then I know that I got less time left. // If I know that it'll (the tumour) spread like wildfire, then I can tell my children and grandchildren that 'now I haven't got much time left. You have to be prepared'. And that's why I want this brutal truth." (Patient; Friedrichsen et al, page 176).	Patients who said that they wanted to hear the absolute truth preferred that because they thought that it was not possible to escape from the truth, as the truth would always be disclosed…it gave a chance to plan their remaining time in a ways that they could choose themselves, for example to prepare their family. (Friedrichsen et al. 2011, page 176).
				"We had a blunt conversation from the start. It was tough, but ultimately made us informed and allowed us to be better prepared for everything to come." (Informal caregiver; Park et al. 2015, page 1474).	Very few respondents in this sample voiced regret that the physicians were honest or clear about their wife's prognosis. Instead, they specifically noted the benefit of honest communication because it allowed them to prepare for end of life. (Park et al. 2015, page 1474).
				"...that is what I've found it hardest to find peace with: did she want to be buried here or did she want to be buried in NN [name of place]?" (Informal caregiver; Røen et al, page 1415).	Two carers got less information than they reported to have wanted and needed, and reported that this negatively affected their preparedness for death and funeral, and their relation to the patient. (Røen et al. 2018, page 1415).
				"He had complete knowledge about his condition and hence was very bold and fully prepared to face his death." (Informal caregiver; Sudhakar et al, page 114).	The knowledge of prognosis was found to cause major effect in the psychological state and preparedness of the patient. (Sudhakar et al, page 114).
				"Psychologically she was very strong and that gave her peace during her final days" (Informal caregiver; Sudhakar et al, page 114).	Few patients were found to accept their states of illness and move forward adaptively, while even preparing themselves to face death. (Sudhakar et al, page 114).
		Loss of hope	1	"[The doctor] is not God so he can't say exactly you have six months. I think he gave away hope. In dad's eyes I can see that he lost a bit of hope." (Informal caregiver; Kirk et al, page 5).	Even in the end stages, patients and families still wanted the door to be left open for the possibility of a miracle; many expressed a compartmentalised awareness, simultaneously acknowledging the terminal nature of the illness, while retaining a need and sense of hope. To have hope dashed
				"I thought, oh well, they have given up on me, I am going to die, even though I	

				knew I was going to die." (Patient; Kirk et al, page 3).	by a rushed or insensitive health carer was experienced extremely negatively. (Kirk et al. 2004, page 4)
		Worry about dying	1	"A female participant (52 yr) described how she used to 'dream of dying and handling it with decorum." (Patient; Barnett, page 52).	Others [patients] worried about dying itself. (Barnett, page 52).
				"Another woman (62 yr) worried about 'how she was going to die and where', but was 'frightened to ask questions' " (Patient; Barnett, page 52).	
				"What will happen at the end; when and how; worried that his son will find him." (Patient; Barnett, page 52).	A particularly poignant fear was expressed by one man (46 yr). (Barnett, page 52).
	Emotional functioning/wellbeing	Avoidance/ denial	6	"I do not want to know. And I told them that right off the bat. No one ever told us much, but when we met with the doctor, it seemed she wanted to reveal to us where we stood, and I interrupted her, and said that I really do not want to, I cannot hear that so please do not share that with me." (Informal caregiver; Applebaum et al, page 820).	Some informal caregivers reported wishing that they had less prognostic information or were resistant it communication with physicians about prognostic information. (Applebaum et al, page 821).
				"Initially when we first came to MSK, she said she could give us the numbers. And (patient) and I both said we didn't want to know." (Informal caregiver; Applebaum et al, page 820).	
				"We have not asked a lot of questions about prognosis with the doctors because of (patient's) desire to not really talk about that part." (Informal caregiver; Appelbaum et al, 820).	
				"I wish I did not know so much sometimes." (Informal caregiver; Applebaum et al, page 820).	
				"We try to avoid looking at the future; we try to live one day at a time, one step at a	

				time. We know how serious it is but we try not to get too caught up in it." (Informal Caregiver; Applebaum et al, page 820).	
				"We treat our life as if despite all of these setbacks that she's going to live a normal lifespan. We haven't made any plans." (Informal caregiver; Applebaum et al, page 820).	
				"We were given statistics and we were asked if we wanted to know more, and we both looked at each other and said no." (Informal caregiver; Applebaum et al, page 820).	
				"Too scared to ask" (Patient; Barnett, page 52).	Another young woman (23 yr) was concerned about her prognosis, but felt that with her rather unusual diagnosis it was difficult to compare with others, and anyway she was 'too scared to ask.' (Barnett, page 52).
				"No, to tell you the truth, I'm very happy that the doctor never made mention of her prognosis. I'm sure the doctor knew because when she [patient] said "How much time have I got," he said, "Well, what do you think?" He left it up to her. He didn't say X amount of time because I think, probably, it would have made her more distraught, and maybe worry more." (Informal caregiver; Cherlin et al, page 1182).	Some caregivers were thankful that the physician did not discuss topics with them...One caregiver expressed not wanting to know more about the patient's illness and life expectancy. (Cherlin et al, page 1181-2).
				"I think maybe the doctor needs to be hopeful, but also plant a seed that [she could be dying]. For instance, he could say, 'We have another drug we can try. I don't know if it will work or not.' But that is not what he did. He didn't say 'I don't know if it will work.' Instead he said, 'I	Some caregivers were ambivalent about how the communication should have taken place and whether they wanted to know more or not. (Cherlin et al, page 1182).

				haven't given up hope on you yet.' May, there is another way to say it. I don't know. Maybe you don't want to hear the news anyways. I don't know." (Informal caregiver; Cherlin et al, page 1182-1183).	
				"No, I don't want to talk about it (the truth)// I think it's possible to suppress this (the disease), maybe years. Not really, but... but a little bit of time. But I don't want to know, and they (the physicians) can't tell you 'whether you'll live two months or one year'. I know that too." (Patient; Friedrichsen et al, page 177).	They [patients] hovered between wanting to know and not wanting to know as they had difficulties in judging the consequences of receiving this knowledge. They weighed pros and cons in an effort to decide whether this information would help them to cope or tip them over into despair. (Friedrichsen et al, page 177).
				"It must be that kind of truth that gives me joy, so to say. Not information that my life soon will be over . Clearly, it must be details that benefit me. It's not that fun to hear that I have a limited time left. I don't think that the truth should be too...well it has to be a little bit modified // I don't want to know, I have never asked if I'll live a full life time, because I don't want to know that." (Patient; Friedrichsen et al, page 176).	Some patients preferred a truth that was not quite the whole truth. They wanted partial truths and particular facts, but not all of the information…hard facts with too detailed information such as having a limited time to live or possibly intolerable symptoms were not included in preferred half-truths. (Friedrichsen et al, page 176).
				"I don't want to know when. I would like to go to sleep and don't wake up, eh, that would be the best thing. I'm not scared, but if somebody is going to say, you've got two days, four days ...I don't want that." (Patient; Kirk et al, page 4).	Patients sometimes verbalised ambiguity: they wanted to be told but they did not want to know. (Kirk et al, page 4).
				"We never talked about death. It was a complete surprise when it happened because we were too focused on the hope of survival." (Informal caregiver; Park et al. 2015, page 1473).	While many men in our sample expressed a wish for more precise information about expected death, this was not universal, and some families appreciated avoidance in the service of hope. (Park et al, page 1475).
				"We never asked how long she had because we didn't want that to effect her	Men described a pattern avoiding discussions of prognosis that was only

				fight. So they did what we wanted." (Informal caregiver; Park et al. 2015, page 1473).<br>"I wish someone had shook me and told me what I need to do. I was in a lot of denial." (Informal caregiver, Park et al. 2015, page 1473).	realised after their wife's death. (Park et al, page 1473).
		Caregiver regret	1	"I wish someone had shook me and told me what I need to do. I was in a lot of denial." (Informal caregiver; Park et al. 2015, page 1473).<br>"I wish I understood how fast the end comes so I could have taken her to Hospice" (Informal caregiver; Park et al. 2015, page 1473).<br>"Maybe we could have changed our discussions from the fight and the forward thinking of what we'll do next, to what needed to be said to each other right then, right when our last few conversations meant the most." (Informal caregiver; Park et al. 2015, page 1474).	Several men commented that their understanding of how much time their wives had left to live impacted treatment decision making near end of life. They described a wish for earlier and more accurate understanding of their wife's prognosis and regretted not utilizing hospice or palliative services sooner. (Park et al, page 1472).
		Distress	1	"And she said I have come to talk to you about palliative care, and he just went into an absolute heap. And of course, that word when you say 'palliative care' he immediately thought death in three months. He just went into absolute shock—burst into tears. . . It was too soon." (Informal caregiver; Kirk et al, page 5).	Although most patient and families requested this information [prognosis] as soon as or shortly after diagnosis was confirmed, many reported that they were distressed as how it was given. Some stated the information has been given too soon or when they hadn't asked for it. (Kirk et al, page 4).
		Frustration	1	A young woman (47 yr) felt she had 'still lots of things she wants to achieve'. (Patient; Barnett, page 51).	Some [patients] expressed a general sense of frustration at time running out. (Barnett et al, page 51).
		Having the opportunity to say goodbye to loved ones	1	"Would have been nice to know the timeframe sooner. Towards the end everything happened so fast we never got to say goodbye to each other."	Several men specifically noted that neither they nor their children had the opportunity to say good-bye to their wives due to misunderstanding about how much time

				(Informal caregiver; Park et al. 2015, page 1473).	they had left with them. (Park et al, page 1474).
	Social functioning	Patient-caregiver relationship	1	"We haven't talked about death, for example […] And not having those kind of talks has affected our relationship […]" (Informal caregiver; Røen et al, page 1415).	Two carers got less information than they reported to have wanted and needed, and reported that this negatively affected their preparedness for death and funeral, and their relation to the patient. (Røen et al, page 1415).
		Communication between patient and family/friends	1	"They [family] know anything I know…what I haven't done is asked them, you know, what's going to happen…I think they've taken it, in a sense, a lot harder than me…all I'm going to have to do is die. They've got to watch me die and then get on without me" (Patient; Kirk et al, page 6).	Patients and family members did not talk as openly and sheltered each other from knowledge. (Kirk et al, page 5).
				"The conversation it pretty open between us. [A few moments later in the interview] I believe that she doesn't acknowledge the extend that they changes are happening" (Informal caregiver; Kirk et al, page 6).	
	Global quality of life	Quality of life	1	"Her oncologist said to her, 'I want to continue with the treatment, there's a 30% chance here.' He has not ever said a 30% chance of what or for how long, but just hearing that has been what has kept her quality of life for these past six months so much more bearable and better than without hearing that." (Informal caregiver; Kirk et al, page 5).	Even in the end stages, patients and families still wanted the door to be left open for the possibility of a miracle; many expressed a compartmentalised awareness, simultaneously acknowledging the terminal nature of the illness, while retaining a need a need and sense of hope. (Kirk et al., page 4).
	Delivery of care	Patient-doctor relationship	1	"He set everything up to see [patient]…then he said 'by all means, come back…we are not going to forget about you. We don't want you lost between the cracks,' were his exact words. They were super, great people" (Patient; Kirk et al, page 3).	The extent to which messages given by the healthcare provider indicate that they will not abandon the patient/family as the illness progresses (Kirk et al, page 3).

				"It was so hard because I felt a little betrayed by [the doctor]. I had trusted him and we had forged a relationship over a number of months ...he hasn't been to see me or talk to me since." (Patient; Kirk et al, page 3).	
		Treatment preferences	4	"Well, none of us would have made the decisions we did [to continue treatment] if we had known the truth about her illness. I just don't know if the doctors knew, but they must have. Why wouldn't they tell us? You have got to wonder why they put her through all that—I mean the chemo and especially the radiology and all those burns. She was in pain and had burns everywhere from the radiation. It was awful. She wouldn't have gone through it if she had known what they knew, but they told us it was curable; so what are you going to do?" (Informal caregiver; Cherlin et al, page 1182).	The following quotations from two caregivers reflect the more common desires of wishing they had been told more by the physicians and believing that they might have made different decisions had they known more (Cherlin et al, page 1182).
				I don't think the decisions were in our control at all. We were not the empowered ones in this because we did not know. How can you know? I mean, we are not the experts in medical things. Should we be? We didn't really have any decisions to make because we didn't know anything. And they told us that her disease was curable. They even said the cancer was gone. That still has me wondering even now. What did they know?" (Informal caregiver; Cherlin et al, page 1182).	

				"Initially they (carers) would like to know what the periods involved are, and I'm talking about people who may be working, who really haven't got unlimited time and it may help them make a decision about how to care for the person, whether to care at game or whether to have it done at the hospital. Because if you've got other things, and everybody does have other things, you can't put your life on hold for an unlimited period. So I would think they'd need to have some idea of time frames, a rough time frame anyway" (Informal caregiver; Clayton et al, page 737).	Some carers said their reasons for needing a time frame were different to those of the patient; for example, knowing how much time to take off work and whether to call other family members to share the care-giving burden. (Clayton et al, page 736).
				"In the meantime, my dear friends have all got together and said you've got to go the herbal way go and see this dear herbal biologist….he's put me on all this stuff - but it hasn't done much [laughter]" (Patient; Kirk et al, page 6).	Secondary sources expanded information, which decreased uncertainty, allowed the search for hopeful alternatives (treatment options or alternative therapies), gave some sense of control, and helped to make or confirm decisions about care, treatment, or lifestyle choices. (Kirk et al, page 5).
				"He advised me not to even bother going for treatments. We were talking about maybe even going into alternative medicine. And he says, well don't bother with that, it's just a waste" (Patient; Kirk et al, page 5).	
				"We pursued treatment until the end…. I was expecting one more scan result that would prove to both of us that we have tried everything medically possible, thus providing the freedom to pull back from curative treatment. However an infection in her one good lung proved fatal before we got there. I wish we knew that those types of scenarios were a much more realistic possibility at our stage in the fight. But at the same time, looking back,	Several men commented that their understanding of how much time their wives had left to live impacted treatment decision making near end of life…others notes that they would have deferred additional chemotherapy, with several men reporting that their wife received treatment within days to weeks of her death. (Park et al, page 1474).

				I guess I should have known." (Informal caregiver; Park et al. 2015, page 1474).	
		Change in information needs	2	"You should not know more than you can take, so to say. Otherwise you can be really frightened and depressed. // physicians knows exactly, they have seen the last phase of life. I don't think it's necessary for them to tell patients every detail about the future. You can give some doses now and then." (Patient; Friedrichsen et al, page 177).	The previous message about discontinuation of tumour treatment was more than enough. They [patients] wanted to live in a positive frame of mind, to maintain their desirable truth, as this way of thinking helped them to continue living. They felt that physicians should provide information that benefits the patient, not frightening details. (Friedrichsen et al, page 177).
				"At the beginning I needed tons (of information). And then at one point I just got tired, you know…And right now I believe I have plenty of information and I would just like to be left to my own devices for a while." (Patient; Kirk et al, page 6).	Many patients reported not wanting as much detail about prognosis as they has asked for initially. (Kirk et al, page 5).
				"On one hand I would like to [know my prognosis now] ...if I know, I can tell myself, so that's it. But I'm not so interested any more. [It has changed?] Yes" (Patient; Kirk et al, page 6).	
				"What else is there to know, really? There's nothing more to know. I feel, anyway." (Patient; Kirk et al, page 6).	
				"What good does it do to know any more at our stage, ah ...I guess what I concern myself about is, will there be a huge amount of pain and discomfort and suffering as we get near the end, you know, and that sort of thing . . . I'm not fully sure how that will unfold." (Informal caregiver; Kirk et al, page 6).	

				"Now, I guess I would get anxious as to what is going to happen to me as I get worse, and whether I can stay at home and be here, or whether I would have to go to hospital." (Patient; Kirk et al, page 6).	
		Conflicting preferences for prognostic information between patients and caregivers	3	"I have read constantly about the future, but I do not discuss it with (patient). As far as what I have read online and that sort of thing, I know it's very bad. But we do not discuss that directly with her doctors, I'd like to, but I know he doesn't want to know." (Informal caregiver; Applebaum et al, page 820).	Many of the informal caregivers interviewed here reported having and wanting more prognostic information than the patients for whom they were providing care. (Applebaum et al, page 821).
				"Two years ago we had an appointment with the doctor and we discussed on the way down that she wanted to know how long she had to live. And I disagreed with that, I thought it would have been better not to know. But it's her life, I said whatever you want to do is fine with me." (Informal caregiver; Applebaum et al, page 820).	
				"We have not asked a lot of questions about prognosis with the doctors because of (patient's) desire to not really talk about that part." (Informal caregiver; Applebaum et al, 820).	
				"I don't believe she has a lot of time left. No one will actually tell me which is in some ways good. U wouldn't tell, I don't think mum needs to know. She seems to think she has a year of more" (Informal caregiver; Kirk et al, page 6).	In early stages families and patients talked to the health carers together. In later stages family members often talked to them alone, often at the patient's request, and did not confirm the patient's exact state of knowledge…patients focused more on daily living and concerns about managing symptoms; families were more concerned with prognosis and details related to care. (Kirk et al, page 5).

				"So when I was together with her, nobody would break their deal with her (sighs), and personally, what I miss most now, is to have had a talk with NN [name of deceased] about what she actually wanted when she died." (Informal caregiver; Røen et al, page 1415).	Two patients did not want information about short life expectancy, contrary to their carers preferences…healthcare professionals and carers followed the wishes of the patients (Røen et al, page 1415).
				"Our model has been that she sits in the front seat. So she decided, and has decided how we should handle this. And I follow her decisions. But that means that I haven't had that much contact with the health services." (Informal caregiver; Røen et al, page 1415).	
		Having a survival time frame	3	"As I said no-one's god and no-one can say your time's going to be up in 6 months, but I think if you've got some idea…you can put your life in order and get your family and that prepared a bit. I think that's good." (Patient; Clayton et al, page 737).	Some patients and carers said that it was important to them to be given a survival time frame. (Clayton et al, page 736).
				"Initially they (carers) would like to know what the periods involved are, and I'm talking about people who may be working, who really haven't got unlimited time and it may help them make a decision about how to care for the person, whether to care at home or whether to have it done at the hospital. Because if you've got other things, and everybody does have other things, you can't put your life on hold for an unlimited period. So I would think they'd need to have some idea of time frames, a rough time frame anyway." (Informal caregiver; Clayton et al, page 737).	

				"I wouldn't want a time put on it, I just like to know which hill and valley are we going, downhill and up…is the way I'd describe it. So that you know exactly, well you can't know exactly but you know what road you're travelling down and whether it's going to be a long rocky road." (Informal caregiver; Clayton et al, page 737).	Many patients and carers said they did not want to be given a time frame, but wanted a general indication of what to expect in the future. (Clayton et al, page 736).
				"I wonder whether any kind of time frame would be useful…from very short to a bit longer. If you go beyond that longer, after that is just waiting for it to happen, that is what I find…But if there is anything that the person still wants to do in their life, if they have unfinished business, they should start thinking about it, but not a time frame." (Informal caregiver' Clayton et al, page 737).	
				"The danger is that if you put a time frame on it, that person will believe you…the closer it gets the more freaked out they get. That happened to me wife, that is the median, she believed it and was almost counting the weeks away." (Informal caregiver; Clayton et al, page 737).	
				"I think an average would probably be the best, rather than the how long and how short. I think on average how long people live with the illness, because there will always be people out of the average won't there." (Patient; Clayton et al, page 738).	Those patients and carers who wanted to be given a time frame mostly said they would like to know how long the average person with their condition would live and/or be given a rough range. A few said they would like to know the longest possible time though might live. (Clayton et al, page 739).
				"I expect the time frames should be expressed in months, generally speaking I guess we're talking months or…if someone is going to live for some months, you can indicate that it won't be	

				a year, and then they know that it's within that guide line… so … a parameter which they can think about." (Informal caregiver; Clayton et al, page 738).	
				"Like if they would have told her that in the beginning, they would have given her a time frame, I think that would have been harder. I think now that she has dealt with many things, come to terms with a lot of things - this is just the final part of that whole circle" (Informal caregiver; Kirk et al, page 4).	Although most patients and families requested this information [about prognosis] as soon as or shortly after diagnosis was confirmed, many reported that they were distressed at how it was given. (Kirk et al, page 4).
				"Would have been nice to know the timeframe sooner. Towards the end everything happened so fast we never got to say goodbye to each other." (Informal caregiver; Park et al. 2015, page 1473).	The desire to understand the 'timeframe' at the end of their wives' lives was a source of impassioned responses for many of the men, with some specifically stating that they relied upon their wife's physician for this information. (Park et al, page 1474).
		Needing additional information	3	"I am interested in understanding his prognosis, I believe that information exists, it's just a matter of me sitting down to read and understand." (Informal caregiver; Applebaum et al, page 820).	Informal caregivers reported desiring more information than they had been given by physicians. This desire may have led informal caregivers to rely on more alternative information sources such as the internet. (Applebaum et al, page 821).
				"I'd like to be more aggressive in getting information." (Informal caregiver; Applebaum et al, page 820).	
				"When he was first diagnosed I read everything that I could find on his condition." (Informal caregiver; Applebaum et al, page 820).	
				"I go to the library over here, on the internet, and I also get a lot of information from my brother." (Patient; Kirk et al, page 5).	Most patients and especially family members obtained additional information from the internet, friends, support groups, books, or second opinions from other health providers, conventional or alternative. (Kirk et al, page 5).
				"The doctors don't have time to sit there and explain every treatment that's available so, I believe, if you want to be informed you've got to do your own	

				homework, yes." (Informal caregiver; Kirk et al, page 6).	
				"So she [daughter] knows that by going on the internet she's got the actual information we're supposed to have." (Patient; Kirk et al, page 6).	
				"In the meantime, my dear friends have all got together and said you've got to go the herbal way go and see this dear herbal biologist . . . he's put me on all this stuff—but it hasn't done much [laughter]." (Patient; Kirk et al, page 6).	
				"One of my son's best friends is a GP . . . And we would sort of check back with him, are we getting the right sort of stuff. We downloaded from the internet information on bisphosphonate." (Patient; Kirk et al, page 6).	
				"…maybe it was our naivete but we sometimes felt like we were kindergarteners tossed into high school, and everyone expected us to know what was going to happen…we didn't know, and trying to figure what questions to ask was really tough, and not to mention a little disheartening." (Informal caregiver; Park et al. 2015, page 1474).	Thirty-three percent of respondents believed that they needed more information about what to expect about their wives' disease courses, death processes, potential treatment complications, and palliative and hospice care. (Park et al, page 1474).
				"They were very open and honest about her prognosis, but really didn't prepare us for how things would progress and what to expect as the disease progressed" (Informal caregivers; Park et al. 2015, page 1473).	
				"Without a question, the single most difficult thing for me was that I had all the resources lines up that I could possibly think of - medication, home care, hospice, etc., and there was not one	

				single thing that was offered to me as the caregiver as to what I could use as support for me and my children." (Informal caregiver; Park et al. 2015, page 1473).	
	Perceived health status	Being aware of prognostic uncertainty	3	"I think it's an embarrassment for the patient to ask the professional how long have I got. I mean they have got no way of diving, you know the future. And I have never asked." (Patient; Clayton et al, page 737).	Most patients and carers were aware of the uncertainty involved in predicting a person's life expectancy. A few patients even said that it was unfair to ask doctors how long they might live because the doctor cannot possibly know the answer. (Clayton et al, page 736).
				"When it finally happened it was a bit of a shock. I wish they were clearer with me." (Informal caregiver; Park et al. 2015, 1473).	The need for honesty and clarity pf communication by physicians were the most described characteristics of prognostic communication reported by the surviving spouses in our sample. (Park et al, page 1473).
				"We were never given any indication that my wife was going to die…Only after her death, when I questioned him did he acknowledge that his prognosis of a cure had changed and he was just hoping to put her into remission." (Informal caregiver; Park et al. 2015, page 1473).	Those who described their wife's death as happening suddenly were more likely to also describe unclear or misleading communication with physicians. (Park et al, page 1474).
				"They were very open and honest about her prognosis, but really didn't prepare us for how things would progress and what to expect as the disease progressed" (Informal caregivers; Park et al. 2015, page 1473).	
				"Her doctors always tried to stay positive but never truly came out and said she was going to die. They repeatedly told us they'd know when we got to that point. That talk never really came. We stayed upbeat and positive until the end. In retrospect, I'm still unsure if knowing for	Several respondents recognized the difficulty in balancing clear and honest communication with the need for hope, many acknowledging their own ambivalence about which they preferred. (Park et al. 2015, page 1474).

				sure or holding strong hope would be better" (Informal caregiver; Park et al. 2015, page 1474).	
				"They must be clear and honest and say how things are. I find that important." (Informal caregiver; Røen et al, page 1415).	Most carers reported overall to have received direct and honest information about the seriousness of the patients' illness. (Røen et al, page 1414).
		Prognostic awareness	1	"He had complete knowledge about his condition and hence was very bold and fully prepared to face his death." (Informal caregiver; Sudhakar et al, page 114).	Although majority of the patients have not revealed their prognosis by either the caregivers or health professionals, they seemed to suspect the poor prognosis with their worsening physical condition. (Sudhakar et al, page 114).
				"He was aware of his prognosis. But during the final week he slipped into depression suddenly and the final two days were the worst" (Informal Caregiver; Sudhakar et al, page 115).	
				"He was aware of his diagnosis, but he did not know of the prognosis. He was very much worried about his condition and started fearing that something bad was going to happen." (Informal caregiver; Sudhakar et al, page 115).	Few patients were reported to cope due to the ignorance on the knowledge of the prognosis. (Sudhakar et al, page 115).
				"Although his condition was worsened he still was fully conscious and highly confident that he will get back to normal." (Informal caregiver; Sudhakar et al, page 115).	
				"Psychologically, he was confident and hoping that it will be cured since we did not tell him about his prognosis. But during the last few days, he started guessing that something was wrong although he did not talk to us anything about that". (Informal caregiver; Sudhakar et al, page 115).	Patients who were unaware of their prognosis were reported to transition from being not aware to suspecting their prognosis, as their physical health was not stable and improving. The shift in their behaviour was observed by caregivers, which included settling their responsibilities, discussing roles of the family members after

					the death of the patients, and social withdrawal. (Sudhakar et al, page 115).
		Prognostic understanding	2	"But he doesn't know how to speak in layman's terms, and I have to say well come on, make it so I understand it." (Patient; Kirk et al, page 3).	Participants described their need for information about prognosis, its accuracy, and the importance of being able to refine their understanding about prognosis as the disease progressed…many did not realize how rapidly death could occur and did not have important conversations with their wife as a result. (Kirk et al, page 4).
				"Maybe we could have changed our discussions from the fight and the forward thinking of what we'll do next, to what needed to be said to each other right then, right when our last few conversations meant the most." (Informal caregiver; Park et al. 2015, page 1474).	Many men shared a belief that important conversations were deferred because of inaccurate understanding of prognosis. (Park et al, page 1474).
	Personal circumstances	Getting affairs in order	3	"As I said no-one's god and no-one can say your time's going to be up in 6 months, but I think if you've got some idea…you can put your life in order and get your family and that prepared a bit. I think that's good." (Patient; Clayton et al, page 737).	Most healthcare professionals, patients and carers stated that it was important that patients be informed that their lifespan was limited by their disease so they could make plans for the future. (Clayton et al, page 736).
				"It's important to know what's going on. If it's a short time, well, let us know so we can make some plans and look at making the last little bit as good as possible." (Informal caregiver; Kirk et al, page 4).	The need for control as a means of coping that may or may not be met by prognostic information (Kirk et al, page 4).
				"I would have been dead cross if I hadn't found out, [because] you start to think, are my affairs in order, mine weren't. And it has taken me almost till now to sort of get things all organised." (Patient; Kirk et al, page 4).	
				"She said nobody can tell how long you have. But just to get everything in order. I	

				think it was the right thing to do." (Patient; Kirk et al, page 5).	
				"But anyway it's been quite exciting since . . . getting everything done—wills made and all sort of things ...The way it's deteriorated since [previous month] I sort of feel that I want to be ready." (Patient; Kirk et al, page 6).	
				"Psychologically, he was confident and hoping that it will be cured since we did not tell him about his prognosis. But during the last few days, he started guessing that something was wrong although he did not talk to us anything about that". (Informal caregiver; Sudhakar et al, page 115).	Patients who were unaware of their prognosis were reported to transition from being not aware to suspecting their prognosis, as their physical health was not stable and improving. The shift in their behaviour was observed by caregivers, which included settling their responsibilities, discussing roles of the family members after the death of the patients, and social withdrawal. (Sudhakar et al, page 115).
